# Typho-Malarial Fever

**Published:** 1881-10-20

**Authors:** A. C. Osterman


### TYPHO-MALARIAL FEVER.

BY A. C. OSTERMAN, M. D.

In the March number of the Herald appeared an article from Dr. Rodman, describing the so-called Typho-Malarial Fever, and giving he history and treatment of two or three of his cases. My experience and Dr. Rodman's are so exactly similar that I cannot refrain from asking a little space to give to the profession, what has proved, in my hands, a specific in this disease. It was by the merest accident that I discovered the value of mur. tinct. of iron, the details of which I will not trouble you with, but simply give you the history of three of my cases, in which it effected excellent results.

I. George B., aged 19; previous health good, invasion gradual, general malaise and epistaxis for a week; felt chilly on Dec. 9th; was called Dec. 12th, P. M.; found temperature 105, pulse 120, respiration 35, face flushed, no eruption, tongue furred and dry, abdomen tympanitic, gurgling and pain in right iliac region. Prescribed 60 drops of tincture ferri chlor. every three hours, 10 grains quinia every six hours; fearing a tendency to diarrhœa gave ½ oz. castor oil and 10 drops turpentine. Next morning, temperature 101, pulse 84, respiration normal, tongue clean and moist, bowels moved three times during the night, otherwise had rested well, and, with the exception of slight cinchonism, felt better every way. Reduced quinia to five grains three times a day; treatment otherwise the same. His temperature remained 101 for four days, then gradually, subsided; was convalescent within a week.

II. William G., aged 16; previous health good, invasion sudden, epistaxis and headache, Dec. 20th; prolonged chill on the 27th; was called 8 P. M., found temperature 105½, pulse 130, tongue clean, abdomen tender and gurgling in right iliac region, stools liquid, and had four on the 27th. I ordered 60 drops of tincture ferri chlor. every three hours, 10 grains quinia every six hours. Dec. 28th, A. M., temperature 104, pulse 130, diarrhœa ceased, but he had slight dilirium during the night; increased iron to 90 drops. 8 P. M., temperature 102½, pulse 96. Dec. 29th, A. M.. temperature 201, pulse 84, tongue moist; diarrhœa ceased; kidneys active. This condition continued until January 3d, when evening temperature was normal, and I dismissed the case, cured.

III. Spellman S., aged 42; previous health poor, invasion gradual, chilliness and diarrhœa for five or six days. April 12th, slight chill followed by high fever; was called April 18th, 2 A. M., found him delirious; could be easily aroused, but had to engage his attention all the time or he lapsed back into the same condition; temperature 104½, pulse 130, respiration 34, tongue dry, cracked and brown in center; diarrhœa, ten or twelve operations daily. Abdomen tympanitic, tenderness and gurgling in right iliac region. Urine scanty, eruption on thorax and abdomen. Slight cough, sputa numular and streaked with blood; chest resonant on percussion; moist rales posteriorly.

*Treatment,*—Tr. ferri chlor. ʒj, every three hours; quinia x grains, every six hours. Diet exclusively milk.

April 19th, 9 A. M. Temperature 102, pulse 112, diarrhœa ceased; had some sleep during the morning, and kidneys more active; no blood in sputa, tongue moist and clearing off.

April 20th. Temperature 101, pulse 96, respiration normal, no diarrhœa, kidneys very active, passed nearly a gallon of water in the last twenty-four hours; gets plenty of sleep, and has some appetite. His temperature ranged between normal and 101 until April 25th, when it became and remained normal. With the history of thirty-two cases before me, I find only one case where it (the iron) gave me any trouble. The urine becoming extremely acid, and the bladder intolerant, which yielded promptly to alkalies and hip baths.—*Med. Her.*

